# Synthesis and Characterization of Durable Antifog Silane–Pyrrolidone Thin Coatings onto Polymeric Films

**DOI:** 10.3390/molecules29050958

**Published:** 2024-02-22

**Authors:** Natalie Mounayer, Taly Iline-Vul, Shlomo Margel

**Affiliations:** Institute of Nanotechnology and Advanced Materials, Department of Chemistry, Bar-Ilan University, Ramat-Gan 5290002, Israel; natali_monayer@hotmail.com (N.M.); iv.taly@gmail.com (T.I.-V.)

**Keywords:** silane–pyrrolidone coatings, Stöber polymerization, polymeric films, antifog coatings

## Abstract

The fogging of transparent surfaces—condensation of water vapor in the air to a small liquid surface at specific environmental conditions—scatters incident light, creating a blurry vision. Fogging presents a significant challenge in various industries, adversely affecting numerous applications including plastic packaging, agricultural films, and various optical devices. Superhydrophobic or superhydrophilic coatings are the main strategies used to induce antifogging to minimize light scattering. Here, an innovative approach is introduced to mitigate fogging by modifying the surface properties of polymeric films, focusing on corona-treated polyethylene as a model. Coatings were prepared in two successive steps: the addition of radical co-polymerization of methacryloxypropyltriethoxysilane and N-vinylpyrrolidone followed by the step-growth Stöber polymerization of the formed silane monomer. The polymeric dispersion was spread on oxidized films via a Mayer rod and dried. Scanning and force microscopy, FIB, XPS, and UV-vis spectroscopy revealed a thin coating composed of cross-linked siloxane (Si-O-Si) covalently bonded to surface hydroxyls exposing pyrrolidone groups. Contact angle measurements, hot-fog examination, and durability tests indicated a durable antifogging activity.

## 1. Introduction

Fogging, a natural phenomenon characterized by the condensation of minute water droplets on solid surfaces at specific environmental conditions, presents a significant challenge in various fields. Its deleterious impact on surface transparency and optical properties arising from the scattering of incident light waves is well-documented and affects diverse applications. Sectors such as packaging, agriculture, medicine, and optics (e.g., lenses, mirrors, and windshields) are notably affected [1,2,3,4,5,6,7,8].

Efforts to address fogging on surfaces have led to the development of various strategies. One approach involves the meticulous regulation of surrounding temperature and humidity to prevent surface water condensation. Superhydrophobic surfaces, despite their effective stemming of wetting and liquid-repellent properties, face limitations due to their complex design, often requiring intricate hierarchical surface topographies [9,10,11,12,13,14,15]. A biomimetic slippery hydrophobic film (PDMS) was recently used for antifogging applications, demonstrating superior antifogging properties compared to flat films [16]. Oil wettability was customized in air, preserving extreme water repellency [17].

Superhydrophilic surfaces offer promise for practical anti-fogging solutions. Such surfaces, treated with a superhydrophilic coating, are characterized by their smooth topography, enabling the formation of a continuous thin water layer [18,19]. This thin layer significantly reduces light scattering, preserving surface transparency [20,21]. Typically, hydrophilic surfaces with contact angles (CAs) below 40° are explored for anti-fogging purposes; they often comprise hydrophilic chemical surface groups such as hydroxyls, carboxyls, and amines [21,22,23,24]. Synergistic chemical patterns on a hydrophilic slippery liquid-induced porous surface (SLIPS) were used for water harvesting [25].

While usually exhibiting micro-flatness, some roughened surfaces also show anti-fogging properties. However, it is important to note that current anti-fogging coatings are primarily limited to laboratory research due to challenges pertaining to real-world stability, non-environmentally friendly materials, and expensive synthetic processes [26,27,28]. Surface modification techniques are commonly employed to introduce specific functional properties to materials such as polymers, metals, glass, and composites [1,6,7]. These modifications encompass alterations in surface chemistry, energy, and roughness, rendering them suitable for diverse practical applications [29,30,31].

In particular, in the realm of polymers, which possess diverse bulk properties, the incorporation of surface modifications has garnered significant interest, bridging the gap between their inherent characteristics and the requirements of specific applications [32]. Techniques such as extrusion, co-extrusion, and surface coating are prevalent in the plastics industry for enhancing surface properties. These methods, although effective, entail challenges such as the waste of surface-modifying additives and the need for plasma and/or corona treatment to activate the polymer substrate prior to coating [33,34,35]. In this context, the present work presents an innovative approach to address fogging by modifying the surface properties of polymeric films, offering a potential solution that combines simplicity, cost-effectiveness, and environmental considerations.

Polyvinylpyrrolidone (PVP) is distinguished for its remarkable qualities, primarily its strong affinity for water. This versatile polymer’s hydrophilic nature makes it an invaluable choice in numerous applications [36]. Its capacity to readily interact with water enhances its solubility and compatibility with aqueous systems, rendering it an effective stabilizer, binder, or carrier for various substances, including pharmaceuticals, cosmetics, medicine, food, and agriculture [37,38,39,40]. PVP’s inherent water-attracting property promotes improved dispersion and dissolution and enhanced bioavailability of active ingredients [41]. Furthermore, it excels in film formation, contributing to its use in coatings, adhesives, and nanomaterial synthesis [42]. Thus, its hydrophilicity and biocompatibility underscore its diverse utility across multiple industries. However, as PVP coatings onto polymeric films are not based on covalent bonding, they are not stable enough and dissolve easily in aqueous conditions.

**Figure 1:**
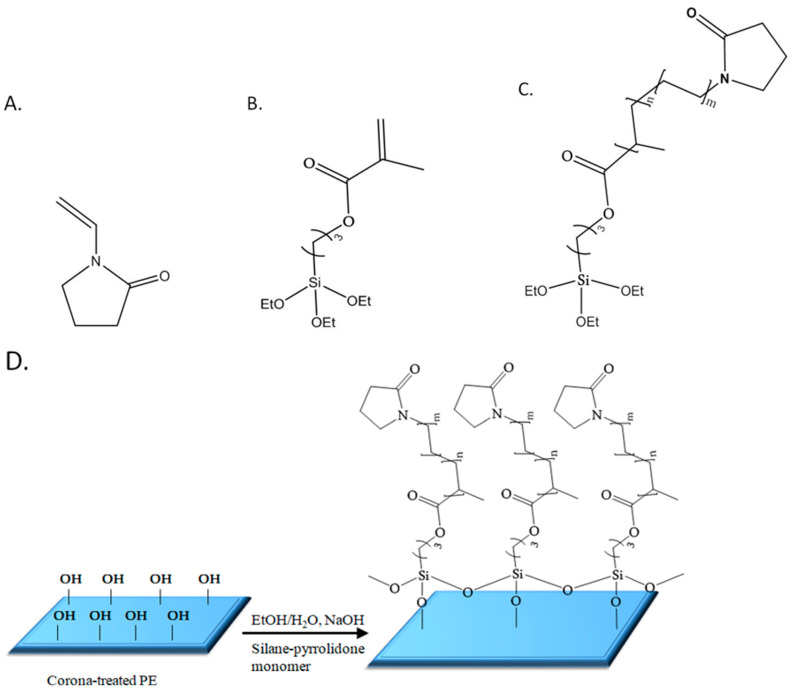
Schematic chemical structures of *N*-vinylpyrrolidone (VP, (**A**)), methacryloxypropyltriethoxysilane (MPTES, (**B**)), and the silane–pyrrolidone monomer (MPTES-VP, (**C**)); schematic illustration of MPTES-VP step-growth polymerization onto the oxidized PE surface (**D**).

**Figure 2:**
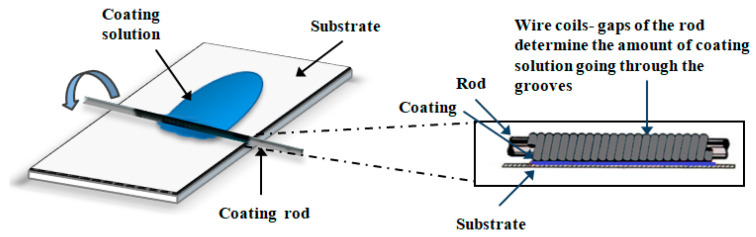
Mayer-rod coating system.

In response to these challenges, a novel method is introduced for achieving durable, thin anti-fogging coatings composed of pyrrolidone groups onto polymeric films such as polyethylene (PE) using a straightforward synthetic approach. Briefly, the coating was prepared in two successive steps: (1) addition co-polymerization in ethanol of *N*-vinylpyrrolidone (VP) (Figure 1A) and the silane monomer methacryloxypropyltriethoxysilane (MPTES) (Figure 1B); (2) step-growth polymerization of the formed silane–pyrrolidone monomer (Figure 1C) via Stöber polymerization [43] followed by spreading of the formed polymeric dispersion on an oxidized film via a Mayer rod and drying. This process forms cross-linked siloxane (Si-O-Si) covalently bonded to surface hydroxyl groups (Figure 1D) [39]. The characterization of the coating was performed using various methods including HRSEM, FIB, ATR, AFM, XPS, CA measurements, UV-vis spectroscopy, hot-fog examination, and durability tests.

## 2. Results and Discussion

### 2.1. Anti-Fogging Poly(MPTES-VP) Thin Coatings onto PE Films

Poly(MPTES-VP) thin coatings onto corona-treated PE films were first prepared through the addition co-polymerization in EtOH of different concentrations (Table 1) of the two monomers MPTES and VP using the initiator AIBN, as described in Section 3.2.1. The formed silane–pyrrolidone monomer was then polymerized in an EtOH/water continuous phase through step-growth Stöber polymerization [43], followed by the spreading of the formed polymeric dispersions on oxidized PE films via a Mayer rod (Figure 1 and Figure 2) to obtain a wet coating thickness of 6 μm, air-drying, and washing with ethanol, as described in Section 3.2.2.

The expected stability of the coating on the surface of the oxidized film is probably due to self-cross-linking siloxane bonds formed between the silane monomer units as well as bonding to the hydroxyl surface groups of the film. It should be noted that surface oxidation of the film was found to be crucial as no significant coating was observed without prior corona treatment. Furthermore, different coatings were synthesized as described in Section 3.2.1 to emphasize the antifog performance achieved by combining the two monomers at specific ratios. 

### 2.2. Contact Angles (CAs)

**Figure 3:**
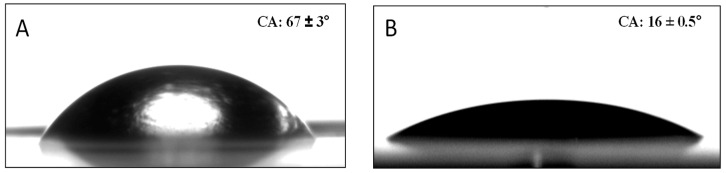
CA images of corona-treated PE (**A**) and PE coated with P(MPTES-VP).1 (**B**).

Water sessile CAs were measured to determine the surface wettability (Table 2). They are influenced by surface roughness and chemistry; rougher surfaces and hydrophobic chemical groups typically lead to higher CAs, while smoother surfaces and hydrophilic chemical groups tend to produce lower angles (Figure 3). Table 2 illustrates significantly lower CAs with PVP compared to MPTES, 18 ± 1 vs. 63 ± 2° (quite similar to 67 ± 3° for PE), reflecting the hydrophilic nature of PVP, particularly due to the pyrrolidone group, compared to the more hydrophobic nature of MPTES. Accordingly, Table 2 illustrates a much lower CA for P(MPTES-PVP).1, 16 ± 0.5°, similar to PA/PVP, indicating a similar surface concentration of the pyrrolidone groups, and only slightly lower CAs for P(MPTES-PVP).2 and 3, 60 ± 2 and 51 ± 1°, respectively. The lower water CAs with an increasing [VP]/[MPTES] monomer ratio reflects the increased wetting due to the higher concentration of pyrrolidone.

### 2.3. Fourier-Transform Infrared Spectroscopy (FTIR)

**Figure 4:**
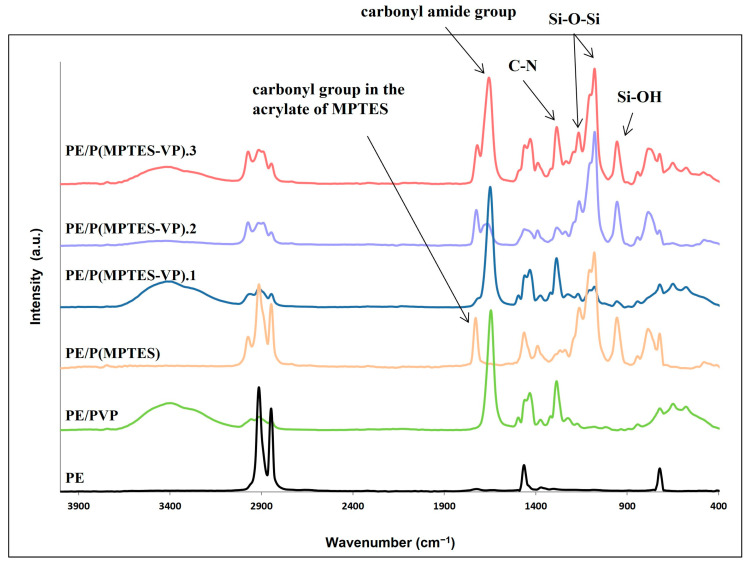
FTIR/ATR spectra of PE and coated films.

FTIR measurements (Figure 4) were performed to ensure the presence of the coating components and demonstrate the change in surface composition. The characteristic peaks of PE at 2850 and 2917 cm^−1^ (C-H asymmetric and symmetric stretching vibrations of methylene groups, respectively), 1473 cm^−1^ (C-H bending), and 731 cm^−1^ (C-C rocking) were clearly observed in the PE spectrum. Figure 4 shows Si-O-Si peaks at 1079 and 1107 cm^−1^ (asymmetric stretching vibration) and a medium band at 954 cm^−1^ (bending of the linear Si-OH stretching vibration) for MPTES and the three MPTES-VP films. As expected, these peaks are less pronounced in MPTES-VP.1 in accordance with the considerably lower concentration of MPTES.

The peaks at 1653 and 1289 cm^−1^ corresponding to the carbonyl amide group and C-N stretching in the pyrrolidone ring [44] are prominent, as expected, in the PVP control and P(MPTES-VP).1, and they decrease at a lower pyrrolidone concentration in P(MPTES-VP).2. As expected, these peaks are not observed in the PE/P(MPTES) spectrum. On the other hand, the peak at 1731 cm^−1^ corresponding to the carbonyl group in the acrylate of MPTES is prominent in P(MPTES) and P(MPTES-VP).2, yet it can hardly be seen in P(MPTES-VP).1 and is not observed at all in the PVP coating.

### 2.4. High-Resolution Scanning Electron Microscopy (HRSEM)

**Figure 5:**
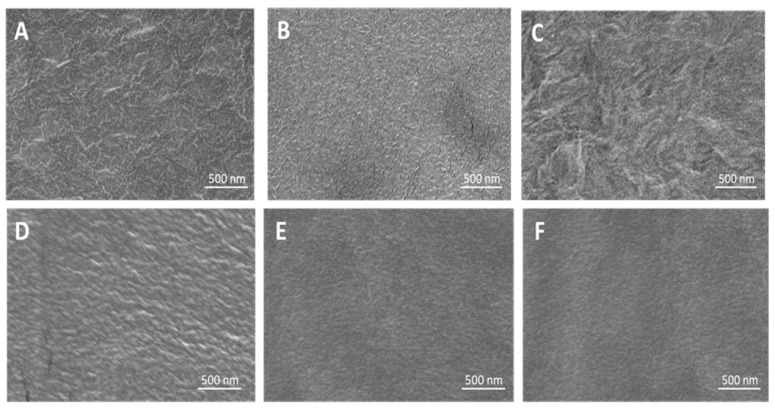
HRSEM images of PE (**A**), PE/P(MPTES-VP).1–3 (**B**–**D**), PE/P(MPTES) (**E**), and PE/PVP (**F**) films.

The HRSEM images (Figure 5) illustrate the surface morphology of PE and the coated films. Smooth surfaces are seen with P(MPTES), PVP, and P(MPTES-VP).1 compared to rougher surfaces of corona-treated PE and PE/P(MPTES-VP).2 and 3 coatings.

### 2.5. Atomic Force Microscopy (AFM)

**Figure 6:**
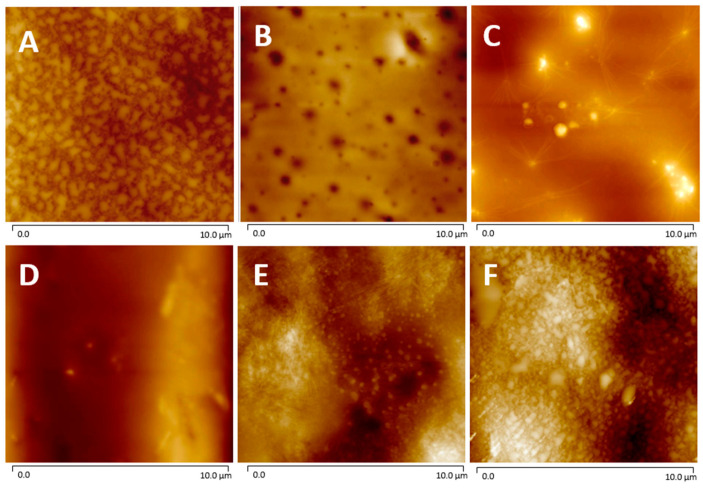
AFM images of PE (**A**), PE/P(MPTES-VP).1–3 (**B**–**D**), PE/P(MPTES) (**E**), and PE/PVP (**F**) films.

AFM measurements were performed to determine the relative surface roughness (Rq) of the non-coated and coated films as shown in Figure 6 and Table 3. The Rq of the corona-treated films was 25 ± 4 nm, while the PVP and P(MPTES) coatings exhibited slightly lower values, 16 ± 4 and 17.6 ± 0.4 nm, respectively. Table 3 indicates that P(MPTES-VP).1 significantly decreased the roughness from 25 ± 4 to 6 ± 3 nm, while P(MPTES-VP).2 and 3 resulted in 23 ± 2 (similar to PE) and 39 ± 8 nm, respectively. These findings align well with HRSEM morphology (Figure 5) and are consistent with expectations. As mentioned above, a low surface roughness is typically used for antifogging surfaces. 

### 2.6. Focused Ion Beam (FIB)

**Figure 7:**
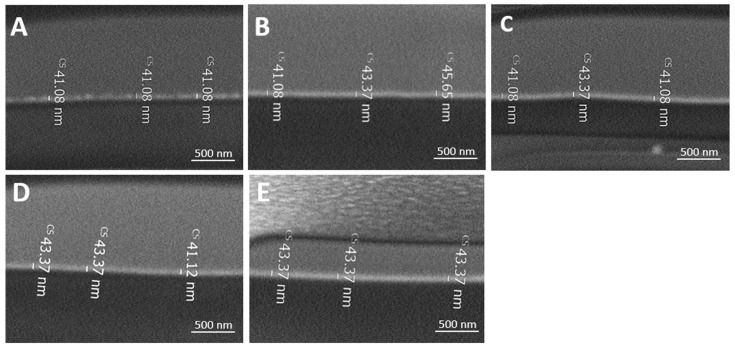
FIB images of P(MPTES-VP).1–3 (**A**–**C**), P(MPTES) (**D**), and PVP (**E**) coated PE films.

The thickness of the various coatings onto the PE films were measured using FIB. Figure 7 and Table 4 illustrate a similar dry thickness between 41 ± 2 and 44 ± 2 nm.

### 2.7. X-ray Photoelectron Spectroscopy (XPS)

**Figure 8:**
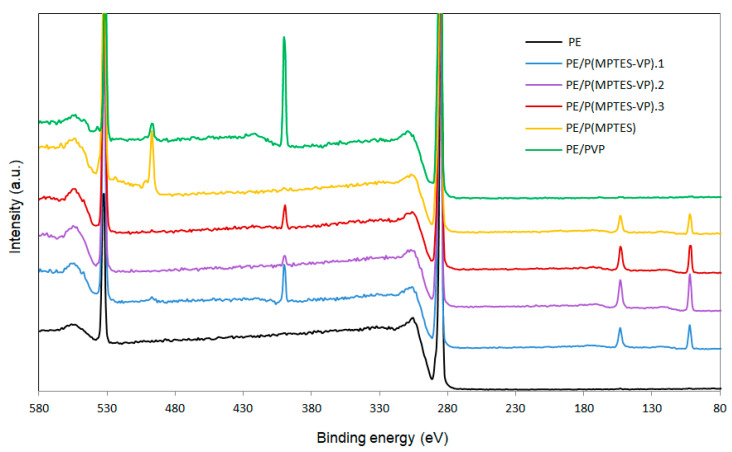
XPS spectra of PE, PE/P(MPTES-VP).1–3, PE/P(MPTES), and PE/PVP films.

Surface elemental analysis was performed to confirm the presence of the thin coating. The XPS spectrum of the corona-treated PE film contained typical peaks of C 1s at 286 eV (87.56%) and O 1s at 532 eV (12.21%), belonging to surface oxidation. The film also contained traces of N 1s at 400 eV (0.23%), which was probably due to environmental contamination (Figure 8 and Table 5) [45]. Table 5 also shows the atomic percentage of Si 2p (102 eV), C 1s, O 1s, and N 1s. The Si surface content of PE/P(MPTES-VP).2, 3, and 1 is 5.3, 4.41, and 3.44 wt%, in accordance with the initial [MPTES]/[VP] ratio used for polymerization (5.0, 1.0, and 0.2).

The very low Si content (0.17%) of the PVP coating was probably due to environmental contamination. Please note that the peak at 497 eV in the spectra of PE/P(MPTES) and PE/PVP (Figure 8) is probably related to the -CN nitrogen of the AIBN initiator [46]. It is hardly observed in the three P(MPTES-VP) coatings, as these polymers were produced in two steps: polymerization of MPTES and VP initiated by AIBN followed by polymerization of the produced silane–pyrrolidone monomer onto the film without AIBN. It should also be noted that the N peak of AIBN is significantly higher in P(MPTES) than in PVP. This may be explained by the higher molecular weight of PVP vs. P(MPTES), so that in each polymeric chain, the concentration of N belonging to the initiator is significantly lower.

### 2.8. UV-Vis Spectroscopy

**Figure 9:**
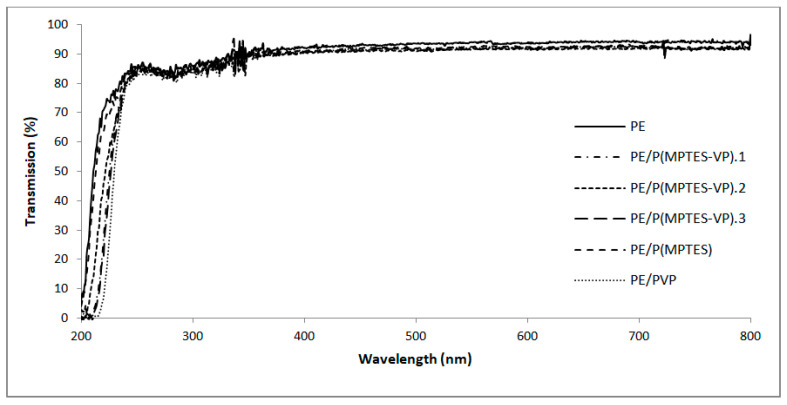
UV-vis spectra of PE and coated PE films.

A high optical transparency is an imperative characteristic in the plastic film industry, particularly in packaging applications. Consequently, visible transmission was measured and compared to an uncoated PE film to determine if the coating influences the transparency. Our analysis revealed that the transmission of UV-visible light, both before and after coating, was not affected, as shown in Figure 9. These findings underscore the outstanding transparency of the coatings, making them well-suited for anti-fog applications in industries such as medicine and food and plastic packaging.

### 2.9. Hot-Fog Tests

**Figure 10:**
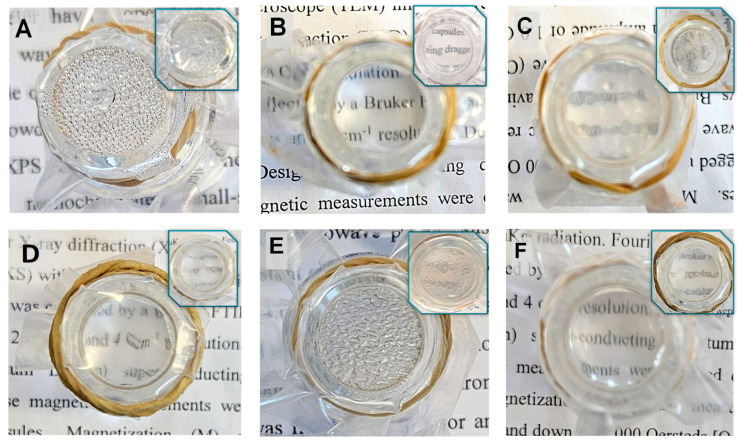
Non-coated and coated PE films after the hot-fog test: PE (**A**), PE/P(MPTES-VP).1 (**B**), PE/P(MPTES-VP).2 (**C**), PE/P(MPTES-VP).3 (**D**), PE/P(MPTES) (**E**), and PE/PVP (**F**). Pictures were taken 1 h after the water in the vial reached 70 °C (images after 24 h are shown in insets).

Hot-fog tests were used to simulate conditions that lead to fogging on the PE surface, as described in Section 3.3.8. The tests were performed on PE films in comparison to films coated with a thin layer. Pictures to visualize the anti-fogging properties were taken 1 h after the water in the vial reached 70 °C. As shown in Figure 10, the film (Figure 10A) showed a foggy surface throughout the experiment. Similar results were observed for P(MPTES) (Figure 10E), except that the water droplets on this film were slightly larger. In contrast, PE/P(MPTES-VP).1 exhibited excellent transparency.

PE/PVP (Figure 10F) demonstrated quite good transparency and slightly lower anti-fog efficiency than PE/P(MPTES-VP).1. However, the anti-fogging properties of PE/PVP decreased over time, so that after 24 h, it became similar to the control film. We assume that this behavior is due to the water dissolution of the PVP coating over time. On the other hand, the PE/P(MPTES-VP).1 film retained its anti-fog performance.

Figure 10 also illustrates that the anti-fogging properties of the P(MPTES-VP) films follow this order: PE/P(MPTES-VP).1 > 3 > 2 (Figure 10B, C, and D, respectively), in accordance with the VP/MPTES volume ratios used for the preparation of P(MPTES-VP)—5.0, 0.2, and 1.0, respectively. The higher this ratio, the better the anti-fogging properties of the film owing to the increased pyrrolidone concentration that results in higher surface energy and increased hydrophilicity, which facilitate the spreading of water droplets on the film surface.

### 2.10. Coating Durability

**Figure 11:**
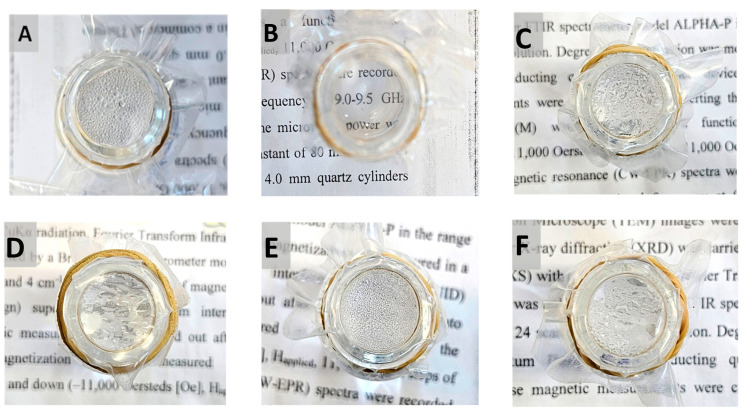
Washed PE films after the hot-fog test: PE (**A**), PE/P(MPTES-VP).1 (**B**), PE/P(MPTES-VP).2 (**C**), PE/P(MPTES-VP).3 (**D**), PE/P(MPTES) (**E**), and PE/PVP (**F**). Pictures were taken 1 h after the water in the vial reached 70 °C.

Durability tests were performed on the best anti-fogging coating, P(MPTES-VP).1 [46]. This includes tape test and washing with ethanol followed by water, as described in Section 3.3.9. These tests aimed to examine the strength of the interaction between the coating and the film and to evaluate its viability for industrial applications. The various dried coatings, following washing with ethanol and water, displayed alterations in their antifogging properties (Figure 11). PE/P(MPTES-VP).1 exhibited no noticeable change in antifog properties during the hot-fog test. Conversely, a deteriorating effect was observed in all other coatings. Therefore, the best antifog film coating, P(MPTES-VP).1, was assessed in further durability tests. 

The tape test was conducted using adhesive tape pressed onto the coating film and then slowly peeling it off and measuring the film’s CA. The tape test was repeated 10 and 20 times to ensure that the anti-fog properties were not damaged; the CA was 16 ± 2 and 22 ± 6°, respectively, similar to the initial value (16 ± 0.5°), illustrating the stability of the coating.

Moreover, FTIR-ATR, AFM, FIB, XPS, and HRSEM measurements after washing with ethanol followed by water revealed no significant alterations, further indicating the durability of the coating.

It is noteworthy to emphasize that the stability of the silane–pyrrolidone monomer solution persists for several months. During this period, polymerization in EtOH/H_2_O followed by spreading on corona-treated PE as described in Section 3.2.1 and Section 3.2.2, yields anti-fog properties comparable to those observed with freshly prepared monomer. These results underscore the chemical durability of the coating attributed to the formation of covalent bonds with functional surface groups of the corona-treated film.

## 3. Experimental Section

### 3.1. Materials

The following analytical grade chemicals were purchased from Sigma-Aldrich (Rehovot, Israel) and used without further purification: absolute anhydrous ethanol (EtOH, HPLC), *N*-vinylpyrrolidone (VP), sodium hydroxide, and methanol. Methacryloxypropyltriethoxysilane (MPTES) was acquired from J&K Scientific Ltd. (Guandong, China). Azobisisobutyronitrile (AIBN, Sigma-Aldrich) was recrystallized from methanol. Double distilled water (DDW) was obtained from a TREION purification system (Tel-Aviv, Israel). Air corona-treated PE films were provided by Poleg Ltd. (Kibutz Gevim, Israel).

### 3.2. Methods

#### 3.2.1. Co-Polymerization of MPTES and VP

A radical polymerization of different concentrations of MPTES and VP to produce the silane–pyrrolidone monomer was performed under a N_2_ atmosphere at 70 °C for 24 h using AIBN as the initiator and EtOH as the continuous phase, as detailed in Table 1.

#### 3.2.2. Preparation of Anti-Fogging Thin Coatings onto Corona-Treated PE Films

Anti-fogging coatings were prepared using a modified Stöber polymerization of the silane–pyrrolidone monomer (prepared as described above) onto the corona-treated PE film with a Mayer-rod coating system (Figure 2) [26]. Briefly, PE films underwent initial surface oxidization via corona treatment (Vetaphone Corona & Plasma, Kolding, Denmark) at 250 W·min/m^2^ to improve the adhesion of the coating on the plastic. Then, 100 μL of 1 mM NaOH aqueous solution was added to 500 μL of coating solution in ethanol. The solution was spread on the oxidized films after mixing with a Mayer rod (No. 2, 4.0 m/min) to obtain a wet deposit thickness (determined by the wire diameter) of 6 μm (RK Print Coat Instruments Ltd., Litlington, UK), left to air-dry, and washed with ethanol.

### 3.3. Characterization of PE Films

#### 3.3.1. Contact Angles (CAs)

Sessile drop water CA measurements were performed using a Goniometer (System OCA, model OCA20, Data Physics Instruments Gmbh, Filderstadt, Germany). DDW drops (5 μL) were placed on four different areas of each film, and images were captured a few seconds after deposition. Static water CAs were determined using Laplace–Young curve fitting. All measurements were performed in the same conditions.

#### 3.3.2. Fourier-Transform Infrared (FTIR) Spectroscopy

FTIR measurements of the non-coated and coated PE films were performed using the attenuated total reflectance (ATR) technique with a Bruker Alpha-FTIR Quick Snap™ (Billerica, MA, USA) sampling module equipped with a platinum ATR diamond module.

#### 3.3.3. Atomic Force Microscopy (AFM)

AFM measurements were carried out using a Bio FastScan scanning probe microscope (Bruker AXS) under environmental conditions in the acoustic hood to minimize vibrational noise. Images were obtained by applying the PeakForce quantitative nanomechanical mapping (QNM) mode with a FastScan-C (Bruker) silicon probe (spring constant of 0.45 N/m) in the retrace direction with a scan rate of 1.7 Hz (512 samples/line). Image processing and roughness analysis were performed using the Nanoscope Analysis 1.5 software by applying the “flatting” and “planefit” functions.

#### 3.3.4. High-Resolution Scanning Electron Microscopy (HRSEM)

Images were taken using a FEI XHR-SEM Magellan 400 L SEM operating at 5 kV. Surface-bonded PE films were imaged to determine the morphology. Samples attached with carbon tape underwent iridium coating under vacuum prior to imaging.

#### 3.3.5. Focused Ion Beam (FIB)

FIB was used to study the cross-section of the coating. The thickness of the surface-bonded layer was analyzed using cross-section images of the film. Cross-sections were prepared using a dual beam system (FIB-SEM) Helios 600 (FEI, Hilsboro, OR, USA), which included an FIB and a SEM column. The Ga^+^ ion beam impacts the sample perpendicularly while the incident electron beam is at a 52° angle to capture cross-section images.

#### 3.3.6. X-ray Photoelectron Spectroscopy (XPS)

XPS measurements for surface elemental analysis of the films were conducted using a Nexsa spectrometer (Thermo Fisher Scientific, Manchester, UK) equipped with a monochromatic micro-focused low-power Al Kα X-ray source (photon energy 1486.6 eV). Survey and high-resolution spectra were acquired at pass energies of 200 and 50 eV, respectively. The source power was normally 72 W. The binding energies of all elements were recalibrated by setting the CC/CH component of the C 1 s peak to 285 eV unless otherwise specified. Quantitative surface chemical analysis was performed using high resolution core-level spectra after removal of a nonlinear smart background. Measurements were carried out under UHV conditions at a base pressure of 5 × 10^−10^ torr (no higher than 3 × 10^−9^ torr). Spectra were analyzed and deconvoluted using Vision 2 Software (Kratos, Manchester, UK). Overlapping signals were analyzed after deconvolution into Gaussian/Lorenzian-shaped components.

#### 3.3.7. Ultraviolet-Visible (UV-Vis) Spectroscopy

UV-vis spectra of the films in the range of 200–600 nm were measured in transmission mode using a Cary 5000 spectrophotometer (Agilent Technologies Inc., Santa Clara, CA, USA).

#### 3.3.8. Hot-Fog Test

Anti-fogging properties were evaluated using the hot-fog test, which simulates fogging conditions [47]. Briefly, a 20 mL vial filled with 5 mL of water was heated to 70 °C. The polymeric film sample was secured to the vial opening with the coated side facing the water. The visibility of the sample was determined through a periodical observation of the water condensing onto the film.

#### 3.3.9. Durability Tests

Durability tests were performed on the prepared coatings to examine the strength of the interaction between the coating and the PE film through a tape test as well as by washing with EtOH and then with water. Tape tests consisted of firmly pressing an adhesive tape onto the coated film and slowly peeling it off as described in the literature [47]. The procedure was performed 10 and 20 times onto the coated film and tested for water CA to ensure that the coating wettability was not damaged. Washing of the coated films was performed by dipping three times (each for about 10 min) in EtOH followed by a similar procedure in DDW and air drying. The thin, coated dry films were examined through FIB, AFM, XPS, and HRSEM, and the best film was tested through hot fog. 

## 4. Conclusions

PVP, owing to its pyrrolidone group, possesses remarkable qualities, primarily strong affinity for water, which enhances its solubility and compatibility with aqueous systems, rendering it an effective stabilizer, binder, or carrier for various substances, e.g., pharmaceuticals, food, and cosmetics [32,33,34,35]. On the other hand, PVP coatings onto polymeric films are not practically used, as these coatings are not covalently bound and, thus, are not stable enough and dissolve easily in aqueous conditions.

Very recently, to overcome the low stability of PVP coatings, Rui et al. prepared relatively stable anti-fog coatings of PVP/silica and PVP/carboxybetaine acrylamide through dip-coating on glass [48]. Here, we illustrate durable anti-fogging thin coatings onto oxidized PE films. The addition polymerization in EtOH of VP with the silane monomer MPTES was followed by a step-growth polymerization of the formed silane–pyrrolidone monomer via Stöber polymerization. The formed polymeric dispersion was spread on the film via a Mayer rod to form the thin antifogging coating on the surface of the polymeric film.

We chose to test three different concentrations of VP and MPTES. However, the best anti-fogging thin coatings were obtained when the [VP]/[MPTES] volume ratio was the highest: 5.0. At this ratio, the wettability properties, as illustrated by water contact angles, of PE/MPTES-VP and PE/PVP were similar, 16 ± 0.5° and 18 ± 1°, respectively. Further studies are ongoing in our lab in order to deepen our understanding of the relationship between surface morphology, roughness, and coating composition on the antifogging efficiency. We plan to extend our study by substituting PE for other polymeric films such as polycarbonate, polypropylene, and polyvinylchloride. Preliminary results on polycarbonate that are currently being evaluated demonstrate a similar activity.

## Figures and Tables

**Table 1:** Polymerization conditions of MPTES and VP (0.2 *w*/*v*% AIBN).

Sample Name	[MPTES] (*v*/*v*%)	[VP] (*v*/*v*%)	[VP]/[MPTES] (*v*/*v*)
MPTES,VP.1	3	15	5.0
MPTES,VP.2	15	3	0.2
MPTES,VP.3	9	9	1.0
MPTES	18	-	
VP	-	18	

**Table 2:** Measured water sessile CAs of non-coated (control) and coated PE films.

Film	PE	PE/P(MPTES-VP).1	PE/P(MPTES-VP).2	PE/P(MPTES-VP).3	PE/PVP	PE/P(MPTES)
CA (°)	67 ± 3	16 ± 0.5	60 ± 2	51 ± 1	18 ± 1	63 ± 2

**Table 3:** Measured roughness (Rq) of non-coated and coated PE films.

Film	PE	PE/P(MPTES-VP).1	PE/P(MPTES-VP).2	PE/P(MPTES-VP).3	PE/P(MPTES)	PE/PVP
Rq (nm)	25 ± 4	6 ± 3	23 ± 2	39 ± 8	17.6 ± 0.4	16 ± 4

**Table 4:** Average FIB-measured thickness of the coatings onto polymeric films.

Film	PE/P(MPTES-VP).1	PE/P(MPTES-VP).2	PE/P(MPTES-VP).3	PE/P(MPTES)	PE/PVP
Thickness (nm)	41 ± 2	44 ± 2	42 ± 1	42 ± 1	43 ± 1

**Table 5:** Quantitative surface XPS analysis of non-coated and coated PE films.

Film	Atomic Concentration (wt%)			
	Si 2p	C 1s	O 1s	N 1s
PE	-	87.56	12.21	0.23
PE/P(MPTES-VP).1	3.44	71.98	20.17	4.25
PE/P(MPTES-VP).2	5.3	69.24	24.25	1.21
PE/P(MPTES-VP).3	4.41	70.33	22.94	2.32
PE/P(MPTES)	3.10	71.80	21.74	0.33
PE/PVP	0.17	75.09	12.57	11.07

## Data Availability

Data are contained within the article.
